# Comparison of the Antioxidant and Sensorial Properties of Kvass Produced from Mountain Rye Bread with the Addition of Selected Plant Raw Materials

**DOI:** 10.3390/foods13030357

**Published:** 2024-01-23

**Authors:** Joanna Kaszuba, Marta Jańczak-Pieniążek, Dagmara Migut, Ireneusz Kapusta, Jan Buczek

**Affiliations:** 1 Department of Food Technology and Human Nutrition, Institute of Food Technology and Nutrition, College of Natural Science, University of Rzeszow, Zelwerowicza Street 4, 35-601 Rzeszow, Poland; ikapusta@ur.edu.pl; 2 Department of Crop Production, Institute of Agricultural Sciences, Environment Management and Protection, College of Natural Science, University of Rzeszow, Zelwerowicza Street 4, 35-601 Rzeszow, Poland; mjanczak@ur.edu.pl (M.J.-P.); dmigut@ur.edu.pl (D.M.); jbuczek@ur.edu.pl (J.B.)

**Keywords:** non-alcoholic cereal beverage, *Secale montamum* L., fermentation, health benefits, UPLC-PDA-MS/MS, black chokeberry, peppermint

## Abstract

Consumers’ growing awareness of healthy nutrition results in an increase in demand for the production of beverages with health-promoting properties. An example of such a product is kvass produced in the fermentation process. This research aimed to determine the impact of plant additives on the antioxidant and sensorial properties of kvass made from bread based on mountain rye flour. The bread extract was fermented at different temperatures (28 and 34 °C). Additives of 3, 5, and 10% were used in the tests, which included black chokeberry juice and infusion, sea buckthorn fruit juice and infusion, and peppermint leaf infusion. A higher fermentation temperature in the production process resulted in an improvement in the organoleptic and antioxidant properties of the tested kvasses. The highest antioxidant activity was demonstrated by kvass with the addition of 10% black chokeberry juice (0.734 µmol Trolox g^−1^ (ABTS), 4.90 µmol of Trolox g^−1^ (DPPH)), and a peppermint leaf infusion (0.773 µmol Trolox g^−1^ (ABTS), 4.71 µmol Trolox g^−1^ (DPPH)). The conditions of kvass production and the type and amount of the additive influenced the selected physicochemical parameters of the obtained kvasses. The chromatographic analysis confirmed the content of 13 phenolic compounds in kvass with the addition of black chokeberry juice, which was 1.68–1.73 mg/100 mL of the finished product with a 10% share of the additive. The 11 phenolic compounds in kvass with the addition of peppermint infusion were confirmed for 7.65–6.86 mg/100 mL of the finished product with 10% of the additive. Kvass enriched with additives from black chokeberry fruit and peppermint leaves may be a promising new category of functional beverages with health-promoting properties resulting from the content of polyphenol compounds. It could be a better base for enrichment with raw materials that are richer in these compounds than pasteurized products.

## 1. Introduction

Cereals are among the strategic groups of crops cultivated worldwide, and since the beginning of civilization, grain has been used for consumption and animal feeding [1]. Cereal grains are an important source of energy, carbohydrates, proteins, vitamins, and minerals. In addition, they are rich in bioactive substances with health-promoting properties. These include phenolic compounds, carotenoids, tocols, and phytosterols, among others, due to which they have high potential as raw material for making plant-based beverages with functional properties [1,2]. Rye is the second most significant cereal, after wheat, whose grain is used to make bread [3], crisp bread, and pumpernickel. The rye grain is rich in fiber (arabinoxylan, β-glucan, cellulose, lignin, and fructan), which helps maintain normal body weight. Fiber also helps reduce the risk of diabetes, heart problems, and cancer [4]. Compared to other cereal species, rye grain has the highest content of bioactive substances, which has a beneficial effect on the human body. These substances include, among others, vitamin E, provitamin A, phytates, and a whole range of other phenolic compounds, including phenolic acids and alkylresorcinols [5,6]. To a lesser extent, the grain of this cereal has found use in the manufacture of fermented beverages such as kvass and alcoholic beverages (whiskey, vodka, and beer) [7]. Due to its low soil and fertilizer requirements, this species can be cultivated in areas with poorer soil, and prone to drought [3,8]. An old species of rye, i.e., mountain rye (*Secale montanum* L.), is worth attention. It is suitable for cultivation in different soil environments and prefers moist, permeable, fertile soil and grows in various soil pH ranges [9]. Due to the growing awareness of consumers about maintaining good health and well-being, there is an increasing demand for products that help improve or maintain both of these. Interest in innovative non-alcoholic and functional cereal drinks is growing rapidly, consistent with the trend of more health-promoting nutrition [10]. New solutions are being sought for food product innovations as well as their manufacturing technologies, including the possibility of enriching grain beverages [2]. An example of such beverages is kvass, which is a traditional beverage in Eastern and Central Europe. Kvass is made through the following two processes: lactic acid fermentation and the alcoholic fermentation of rye raw materials (rye flour, dry rye bread, rye malt), sugar, and yeast. Kvass contains no greater than 1.5% alcohol by volume, while with longer stands, the alcohol concentration can reach 2.5% or more. Unlike other grain-based alcoholic beverages, kvass is considered a non-alcoholic beverage [2,11,12,13,14]. Kvass is a refreshing beverage with a pleasant aroma of rye bread and a moderately sweet and sour taste, which is most in demand during hot weather on summer days [15,16,17]. This beverage does not require additional pasteurization, as the role of preservation of the product is performed using lactic acid formed in lactic fermentation. Kvass contains natural organic acids, amino acids, and carbohydrates, which are important from a dietary and physiological point of view [12]. Kvass shows positive effects on metabolism, contains B vitamins, is a source of folic acid, and helps regulate digestive processes [12,15]. Nowadays, the most common product available in the market under the name kvass is obtained by diluting malt concentrate with a large number of additives, often as a pasteurized product [18]. In recent years, increasing attention has been focused on plants, including herbs, that are rich in polyphenols and exhibit antioxidant activity, which are becoming popular as additives in functional foods and nutraceuticals [19]. An example of such raw materials is black chokeberry (*Aronia melanocarpa*) fruit, which is characterized by a high content of phenolic compounds and is thus counted among the fruits with the highest antioxidant properties. Antioxidant activity includes radical scavenging, the inhibition of the generation of reactive oxygen and nitrogen species, the restoration of antioxidant activity, and the inhibition of pro-oxidant enzymes. In addition, chokeberry fruits are a valuable source of anthocyanins, proanthocyanidins, and hydroxycinnamic acids [20,21]. Sea buckthorn fruits (*Hippophaë rhamnoides* L.), like chokeberries, are a source of biologically active components, which include vitamins, flavonoids, carotenoids, and unsaturated fatty acids, which have a positive impact on human health. Particularly important is the presence of stable vitamin C in these fruits. Sea buckthorn fruits are characterized by a wide spectrum of health-promoting properties due to significant amounts of antioxidant compounds, including flavonoids, tocopherols, and carotenoids [22,23,24]. Peppermint (*Mentha* × *piperita* L.) is mainly grown for its essential oil, which can be extracted from freshly ground leaves, providing a raw material for the extraction of various bioactive compounds [25,26]. In addition to essential oil, peppermint leaves contain the following non-essential components: steroids, flavonoids, triterpenoids, phenolic acids, etc. Thus, this herb is a valuable raw material for the extraction of various bioactive compounds showing anti-inflammatory, antibacterial, antiviral, and anticancer activities [27,28].

There are a few reports in the literature regarding the impact of raw material additives based on black chokeberry fruits, sea buckthorn fruits, and peppermint leaves on the health-promoting properties of kvasses. Given the growing interest in innovative, non-alcoholic, and functional plant-based beverages, research in this area is warranted. For the production of kvass, rye bread was used, made from a raw material other than the commonly used one, which is rye flour from rye grain (*Secale cereale* L.). In this study, rye flour from mountain rye grain (*Secale montanum* L.) was used to bake bread. Accordingly, kvass was produced based on the rye bread extract and enriched with plant additives such as preparations of black chokeberry fruit, sea buckthorn fruit, and peppermint herb. The obtained kvasses with additives were subjected to sensory characteristics, selected quality characteristics, and chemical analyses to evaluate the influence of the form and quantity of the plant additive on the antioxidant of the final product.

## 2. Materials and Methods

### 2.1. Research Material

The material for the study was kvasses made from an extract of mountain rye bread flour. The bread was made using mountain rye bread flour type 720 (Swojska Piwniczka, Zawadka, Poland) with the following composition (g/100 g): fat at 1.3 g, carbohydrates at 70.2 g, protein at 5.6 g, and salt at 0.00 g. For this study, two variants of control kvasses, “28” and “34”, were prepared, as well as kvasses based on them with 5 different additives in proportions of 3, 5, and 10%. The additives were used as follows: BC (I)—added in the form of an infusion of dried and powdered black chokeberry (BC) fruits (Zakład Zielarski KAWON-HURT Nowak Sp. J., Krajewice, Poland), BC (J)—the juice of chokeberry fruits (Premium Rosa Sp. z o. o., Złotokłos, Poland) SB (I)—infusion of dried and powdered sea buckthorn (SB) fruits (NANGA, Blękwit, Poland), SB (J)—the juice of sea buckthorn fruits (Premium Rosa Sp. z o.o., Złotokłos, Poland), P (I)—infusion of peppermint (P) leaves (Zakład Zielarski KAWON-HURT Nowak Sp. J., Krajewice, Poland). Fifteen kvasses were prepared with additives based on each of the control sours, which ultimately accounted for 30 test samples and 2 control samples.

### 2.2. Research Methods

#### 2.2.1. Bread Baking

Dough for bread was conducted using a three-phase method according to Bartnik and Ceglińska [29,30] with modifications. The phases were as follows: the loose leaven phase (300% yield, fermentation time 48 h, fermentation temperature 28 °C), sour phase (200% yield, a combination of sour and flour 1:1, baker’s yeast (LALLEMAND Polska, Józefów, Poland) 1% of the total mass of flour in the dough, phase fermentation time 3 h, fermentation temperature 32 °C), and dough phase (165% yield, combination of acid and flour 1:0.75, table salt 1.5% of the total mass of flour in the dough, fermentation time 45 min, fermentation temperature 32 °C). The fermentation of the sour phase was carried out in a laboratory incubator (Binder GmbH, Tuttlingen, Germany), while the sour and dough phases were fermented in a fermentation chamber (Sveba Dahlen, Fristad, Sweden). After fermentation, the dough was divided into 300 g pieces, which were placed in baking molds and subjected to final fermentation for about 30 min. The molds with the dough were placed in the baking chamber of an electric oven model, Classic (Sveba Dahlen, Fristad, Sweden), heated to 240 °C for 35 min.

After baking and cooling, the bread was stored in LDPE foil bags at a temperature of 20 °C.

#### 2.2.2. Preparation of Control Kvass and Kvasses with Additives

The kvasses were prepared in accordance with the method given by Gambuś et al. [12] with modifications. A portion of 500 g of mountain rye bread, previously chopped into small cubes and air-dried, was added to 1500 mL of water at a temperature of 80 °C, mixed, and left to stand for 30 min, stirring every 5 min. Then, another portion of water (1500 mL) at a temperature of 70 °C was added, stirred, and allowed to extract for 90 min, stirring every 10 min. The mash was then filtered through a sterile gauze, and another portion of water (1500 mL) at a temperature of 70 °C was added to the filtrate. To the filtrate, 100 g of sugar and 20 g of compressed baker’s yeast (LALLEMAND Polska, Józefów, Poland) were added, mixed, transferred to glass bottles (1000 mL), and left to ferment at a temperature of 28 °C (laboratory incubator). Similarly, a second portion was prepared, which was left to ferment at 34 °C (laboratory incubator). The fermentation time for both variants was 24 h. The next step was to separate the resulting kvass from the precipitate via filtration through a sterile gauze. Sugar was added to the collected filtrate at a rate of 10 g/L of liquid, mixed and transferred into glass bottles (1000 mL), closed with twist-off caps, and left in a refrigerator at 10 °C for 14 days. After this time, a portion of kvass was prepared, and selected plant-based additives were added; the final sample volume of kvass with addition was 300 mL. Juices were added by measuring the desired volume from the product package. The infusions were prepared at a ratio of 5 g of plant material per 100 mL of hot water, which was brewed for 5 min, drained, and used to prepare the kvass after cooling (to ambient temperature). The control and tested kvasses were left in a refrigerator at 10 °C for 7 days. After this time, they were subjected to organoleptic and quality tests and also to chemical analysis.

Control kvasses were made in three replicates, and all kvasses with additives were made in each replicate, which constituted three production batches of research material.

#### 2.2.3. Organoleptic Evaluation of Kvasses

The organoleptic evaluation of kvass was performed by 10 trained panelists according to the criteria based on Gambuś et al. [12] with their modifications (Table 1). All tested recipe variants of kvass with additives and control kvass from each production batch were assessed.

#### 2.2.4. Measurements of Antioxidant Activity of Kvasses

Samples of kvasses for antioxidant activity testing were centrifuged for 10 min at 7800 rpm in a centrifuge model 5430 (Eppendorf, Hamburg, Germany); the supernatant was collected and analyzed using a spectrophotometer UV-VIS UV5100 (Metash, Shanghai, China). In spectrophotometer cuvettes, 3 mL each of the ABTS solution (absorbance equal to A = 0.700 nm) was taken, and 0.03 mL each of the prepared supernatants was added. The contents of the cuvette were mixed and incubated for 6 min in the dark. Absorbance was then measured at λ = 734 nm [31]. DPPH assays were also performed. To 1 mL of 100 µM of a free radical solution of 2,2-di(4-tert-octylphenyl)-1-picrylhydrazyl (DPPH-) was 30 µL of the supernatant added. After 30 min of incubation in the dark, the absorbance was measured at λ = 515 nm [32]. The results are given as µmol Trolox g^−1^. The determination was performed in three replicates for each sample tested.

#### 2.2.5. Measurements of Selected Quality Parameters of Kvasses

The measurement of the color parameters of the kvasses was carried out using an UltraScanVIS spectrophotometer (HunterLab, Reston, VA, USA) according to the method described by other authors [33,34]. The color parameters were measured using the CIE L*a*b* system. In this system, L* values represent color brightness and range from 0 (black) to 100 (white), a* values represent green (−) to red (+) coordinates, and b* values represent the proportion of blue (−) to yellow (+) coordinates. The measurement was carried out in 3 repetitions for selected types of tested kvasses from each production batch. Based on the results, the value of the total color difference ∆E = √((∆L*)^2^ + (∆a*)^2^ + (∆b*)^2^) was calculated [35].

The pH value of selected types of kvass was also measured using a laboratory pH meter (Elmetron, Zabrze, Poland) at a temperature of 20 °C ± 0.1. The measurement was carried out in 3 repetitions for the selected types of kvasses tested from each production batch. The alcohol content in the given kvass was determined according to PN-A-75101-09:1990 [36] using the titrimetric method. A portion of 5 mL of kvass and 150 mL of water were distilled and collected in a flask with 25 mL of potassium dichromate (0.2 N) and 25 mL of sulfuric acid (50%). Distillation was carried out until 100 mL of the distillate was obtained. After closing the flask, it was left to stand for 1 h, then 0.5 g of KI was added and titrated with sodium thiosulfate solution (0.1 M) in the presence of an indicator of 1% soluble starch solution until the navy-blue color disappeared.

#### 2.2.6. Chromatographic Analysis of Kvasses by UPLC-PDA-ESI-MS/MS Method

Samples of the control and tested kvass were centrifuged for 10 min at 7800 rpm in a model 5430 centrifuge from Eppendorf (Hamburg, Germany). A clear liquid (supernatant) from the centrifuged solutions was poured off the precipitate. To separate phenolic compounds from the resulting extract, the solid phase extraction method and Sep-Pak-type columns (Waters, Milford, MA, USA) were used. First, the column was conditioned, and then the sample was introduced into the column’s stationary phase. After the extract was introduced, the stationary phase was washed with distilled water to remove impurities and dried. From the Sep-Pak prepared using this method, the analyte was eluted with methanol, which was then evaporated under reduced pressure via a rotary evaporator Rotavapor model R-200 (BUCHI, Flawil, Switzerland) and a Vac model V-500 vacuum pump (BUCHI, Flawil, Switzerland). The remaining analyte precipitate was dissolved in a methanol/water mixture (50:50, *v*/*v*). The dissolved precipitate was quantitatively transferred to Eppendorf-type tubes and centrifuged for 10 min at 10,000 rpm (centrifuge model 5430, Eppendorf, Hamburg, Germany). The separated supernatant was collected and destined for chromatographic analysis.

Polyphenolic compounds were analyzed based on the technique of ultraperformance liquid chromatography coupled to mass detection using an ACQUITY instrument (Waters, Milford, MA, USA) following the methodology described in the study by Kapusta et al. [37]. The chromatograph was equipped with a tandem quadrupole (TQD) mass spectrometer with an electrospray ionization (ESI) source operating in the positive/negative ion sweep mode. The separation of phenolic compounds was carried out using a BEH C-18 UPLC column with a grain size of 1.7 µm and dimensions of 100 mm × 2.1 mm (Waters, Milford, MA, USA). A 0.1% solution of formic acid in water *v*/*v* (eluent A) and a 40% solution of acetonitrile in 0.1% formic acid in water, *v*/*v* (eluent B) were used as the mobile phase. The following linear gradient was applied starting from initial conditions of 20% B and 80% A to 100% B and 0% A within 8 min. The total analysis was the time of 9.5 min at a flow rate of 0.35 mL/min. The sample injection volume was 5 µL, and the column temperature was set at 50 °C. The ESI source was operated at the following ionization parameters: the sampling cone voltage of 30 V, capillary voltage of 3.5 kV, source and desolvation temperatures of 120 °C and 350 °C, respectively, and a desolvation gas flow rate of 800 L/h. Waters MassLynx software v.4.1 was used for data acquisition and processing. 

The characterization of individual phenolic compounds was carried out based on retention times, mass-to-charge ratios, pseudomolecular and fragment ions, and a comparison of the obtained data with data from the literature. Polyphenolic compounds were identified and characterized by comparing UV-Vis absorption maximum spectra, molecular weight determined from the mass-to-charge ratio, retention times, and fragmentation spectra with data from the literature for the samples of kvass with black chokeberry addition and for the samples of kvass with peppermint addition. 

Decay spectra were obtained using collision-induced fragmentation (CID) in a tandem arrangement. The collision energy was selected individually for the compound analyzed.

### 2.3. Statistical Analysis

Statistical analysis was conducted using TIBCO Statistica 13.3.0 (TIBCO Software Inc., Palo Alto, CA, USA). To verify the normality of the distribution, the Shapiro–Wilk test was applied. The results obtained were subject to single-factor analysis of variance (ANOVA). A two-factor analysis of variance (ANOVA) was performed to determine the effect of additive and incubation temperature in making kvass. To determine the significance of differences between the mean values of the parameter studied, Tukey’s post hoc test (*p* ≤ 0.05) was used.

## 3. Results and Discussion

### 3.1. Organoleptic Evaluation and Antioxidant Properties of the Tested Kvass

Control kvasses were obtained at fermentation temperatures of 28° and 34 °C and the tested kvasses based on them with plant additives were subjected to organoleptic evaluation, and the average results are presented in Table 2.

A comparison of the results of tests of individual sensory attributes of the tested kvasses, as well as total points, shows that the use of different additives, both in terms of botanical origin, the form of the additive, as well as participation in the recipe of the product resulted in the significant differentiation of the test results. Kvasses with additives based on sea buckthorn fruit were rated the lowest in terms of the most sensory attributes, while the lowest ratings for product color were recorded in studies of kvasses with additives of dried black chokeberry fruit infusions averaging 2.1 points (FT 28 °C and FT 34 °C) and sea buckthorn fruit infusions averaging 2.0 points (FT 28 °C). An important determinant of the quality of kvass is its taste. In the present study, it was noted that the use of the sea buckthorn fruit juice additive resulted in a significant deterioration in the evaluation of this sensory attribute, which was significantly lower than the results recorded for the control sample and other kvasses with additions. Importantly, it was noted that the evaluation of this parameter in the case of kvasses with additives based on sea buckthorn fruit was influenced by the fermentation temperature of the control kvass, which was used to make the acid with additives, and so the kvasses based on the control acid after fermentation at 28 °C were rated worse. The best and similarly rated flavor of the product with the addition of chokeberry fruit juice and with the addition of peppermint infusion was an average of 4.8 points. These results were not differentiated by fermentation temperature. The sensory evaluation results, both of the control kvasses and the kvasses tested with additives, were higher for products obtained via fermentation at 34 °C. Based on the total points, it was found that the kvasses with added chokeberry fruit juice ranked best in the organoleptic evaluation, scoring better (18.6–19.9) than the control kvass (15.1–17.4), and the kvasses with peppermint infusion averaged 13.2 (FT 28°) and 17.3 (FT 34 °C), which scored close to the corresponding control sample (Table 2). As in the study by Gambuś et al. [12] on the production of kvasses based on a bread extract with the addition of sugar, an unsatisfactory color (kvasses with the addition of infusions) and the low clarity of the products (kvasses with the addition of infusions and sea buckthorn fruit juice) were also noted in the kvasses under discussion (Table 2). In the studies of other authors [12,18] on the use of additives in the production of kvasses, it was noted that in addition to an intended improvement in the chemical composition of the finished product using ingredients with antioxidant properties, there was a significant effect of additives on some sensory characteristics of kvasses. 

The control kvasses and the recipe variants derived from them were subjected to an evaluation of antioxidant properties. Experimental factors and the interaction between them had a significant effect on antioxidant activity measured using both the ABTS and DPPH radical methods (Table 3 and Table S1). 

The higher fermentation temperature (34 °C) increased the antioxidant activity of the control kvass as well as the tested kvasses based on it. When the results were compared using the ABTS radical method, higher values were recorded in comparison with the kvasses obtained via fermentation at a temperature of 28 °C by 12.0% and 16.6% for the DPPH radical method. Additives with health-promoting properties differentiated the antioxidant activity of kvasses. The lowest value of the parameter in question was shown in kvass with 3% black chokeberry fruit infusion (ABTS and DPPH) and 3% sea buckthorn fruit infusion (ABTS). These values were significantly lower compared to the control sample by 65.0, 71.4, and 59.1%, respectively. When measured using the ABTS radical method, smaller values than the control were additionally shown by the kvasses with black chokeberry fruit infusion (5 and 10%), sea buckthorn fruit juice (3%), and sea buckthorn infusion (5 and 10%). However, when measured using the DPPH radical method, lower values compared to the results of the control kvass were shown for kvasses with the addition of black chokeberry fruit infusion (5 and 10%), sea buckthorn fruit juice 3% and sea buckthorn fruit infusion (3 and 10%). The highest antioxidant activity among all the tested kvasses and formulation variants was shown in kvass with 10% peppermint infusion (ABTS and DPPH). In comparison with the control sample, an increase in antioxidant activity by as much as 192.8% (ABTS) and 157.4% (DPPH) was found. High antioxidant activity, confirmed by the measurements used in both methods, was also recorded for kvasses with the addition of black chokeberry fruit juice (5 and 10%) and peppermint infusion (5%). Similarly, the effect of the formulation, especially with the participation of biologically active ingredients, on increasing the antioxidant potential of the ready-made product was also noted by other authors [12,18].

Statistical analysis of the results of the sensory characteristics and antioxidant activity of the kvasses served as a criterion for selecting recipe variants for further study. Kvasses with the addition of black chokeberry fruit juice at 5 and 10% and kvasses with the addition of peppermint infusion at 5 and 10%, which were made based on both control kvasses, were selected for further research. 

### 3.2. Comparison of the Quality Parameters of Selected Tested Kvass

Selected variants of kvasses with additives and control kvasses were subjected to tests of selected quality characteristics. The results are shown in Table 4.

The pH value of the tested control kvasses did not vary and averaged 4.15, while a similar value was also obtained in tests of kvass with 5% black chokeberry fruit juice. In the studies of other recipe variants of kvasses (Table 4), including the participation of peppermint infusion, higher values of the particular parameter were recorded in the range of pH = 4.2–4.6. Similar results were obtained by other authors [11,12,13] in studies of kvass based on rye bread, which confirms that the mountain rye bread used in the discussed studies is also a good raw material for obtaining water extract for fermentation into kvass.

The presence of alcohol (Table 4) was found in the tested kvasses in concentrations not exceeding 0.5%, and its content was lower in products in which part of the kvass was replaced with a peppermint infusion. As can be seen from a comparison of the results of other authors’ studies, the alcohol content of a finished product such as kvass depends on the recipe and the conditions for making the kvass. Of particular importance is the proportion of sugar and the composition of the microflora [11,12,13,18].

The color parameters of the control kvasses (FT 28 °C and FT 34 °C) did not differentiate, while they were subject to the significance of the proportion (Table 4). In the case of kvasses with both additions of chokeberry fruit juice and peppermint infusion, a darkening in the color was noted the greater and the higher the share of the additive and when the chokeberry fruit-based additive was used. There was no effect of the fermentation temperature of the control kvass on changes in the color brightness (L*) of products based on their use with additives. Kvasses with the addition of black chokeberry fruit juice (5 and 10%, FT 28 and 34 °C) had a higher proportion of the red-blue color (10%, FT 34 °C). The direction of changes in the chromaticity coordinates of kvasses with the addition of peppermint infusions compared to the control sample was the opposite. It was noted that the use of peppermint leaf-based infusion resulted in a shift in color toward green and an increase in the proportion of the yellow color. For both recipe variants with the addition of black chokeberry fruit juice and peppermint infusions, the values of chromaticity parameters a* and b*, in addition to the direction of the red/green and blue/yellow color shift, were significantly affected by the share of the additive used and the pH value of the final product.

To objectively assess the difference in color between two objects, the parameter of total color difference, ΔE, was used. To classify differences in perceived color, we used the criterion given by Adekunte et al. [38] as follows: very distinct (∆E > 3), distinct (1.5 < ∆E < 3), and small difference (∆E < 1.5). The color of the tested kvasses with additives (Table 4) was clearly different from that of the control samples. 

### 3.3. Chromatographic Analysis of Selected Tested Kvass

The health-promoting potential of the tested kvass with selected plant additives was assessed based on the results of the chromatographic analysis of the polyphenolic compounds using the UPLC-PDA-MS/MS method (Table 5 and Table 6).

Two anthocyanins and other eleven phenolic compounds were indicated in kvass with the addition of black chokeberry juice (Table 5). Chlorogenic acid (36.2% of total phenolic contents) and neochlorogenic acid (23.5% of total phenolic contents) were found in the highest concentrations. The total content of the determined compounds amounted to an average of 895 µg/100 mL in products with a 5% black chokeberry juice addition, while in kvass with a higher content of this additive (10%), the content was approximately 88.8–92.7% higher and amounted to an average of 1703 µg/100 mL. Other authors [39] showed that chlorogenic and neochlorogenic acids represent the most numerous groups of phenolic acids in the profile of chokeberry polyphenolic compounds. At the same time, the cited authors pointed out the large contribution of cyanidin-3-O-galactoside in polyphenolic compounds. The authors proved that the polyphenol profile of products based on chokeberry fruits depends on the raw material processing method (e.g., drying, pressing).

Even higher contents of selected phenolic compounds were found in the tested kvasses with the addition of a peppermint infusion (Table 6). A higher total content of analyzed compounds in variants, i.e., with the addition of 5 and 10%, was recorded in the tested products based on kvass fermented at a temperature of 28 °C. On average, products (FT 28 °C and FT 34 °C) with a higher proportion of additives (10%) showed a 94.5% higher content of the tested phenolic compounds. Among the eleven identified and quantified phenolic compounds tested, the highest content was found for rosmarinic acid and quercetin 3-O-glucoside-pentoside, which constituted 27.7% and 20.7% (5% of the additive’s share) and 15.6% (10% of the additive’s share), respectively, of the total content of the investigated phenolic compounds in kvasses with the addition of peppermint infusions. The differences in the content and stability of these compounds might be caused by the pH value of the product. In the studies of extracts from peppermint leaves, it was noted that the most abundant ingredients identified were eriocitrin and rosmarinic acid [40]. Numerous studies on the health properties of peppermint leaves [41] prompt plans for further research into the possibility of improving the quality and health-promoting value of kvass enriched with different kinds of products based on peppermint leaves. Similarly, in the above-mentioned studies, other authors also confirmed that the addition of aromatic plant infusions improved the smell, taste, and antioxidant properties of buckwheat kvasses [42].

In studies conducted by other authors [43], chokeberry fruit juice was used to prepare a wort-based drink. The selected optimal concentration of chokeberry juice gave the drink a pleasurable flavor but required some improvements. To solve this problem, peppermint oil was inserted into the mixture, and carbonation was performed using CO_2_ gas. This drink gained greater acceptance when carbonated and mixed with peppermint oil. Similarly to the studies of the cited authors, the results obtained in the discussed studies can be considered promising, and to optimize the quality and health-promoting value of the production of kvass based on a mountain rye bread extract, further research should be carried out.

Other authors [44] also confirmed that enriching typical kvass with fruit- or vegetable-based additives resulted in an improvement in its quality. In the research of the cited authors, jujube–tomato kvass was produced with a unique taste as a rich source of raw materials. It has been reported that the simple production process makes this product suitable for consumption.

## 4. Conclusions

Based on the research conducted on the effect of the application of plant-based additives on the organoleptic and antioxidant properties of kvasses, it was demonstrated that the fermentation temperature of 34 °C during the preparation process improved both of these properties of the tested beverages. Tested beverages with the addition of black chokeberry juice performed best in the organoleptic assessment (*p* < 0.05), obtaining better results (18.6–19.9) than the control kvasses (15.1–17.4), and kvass with peppermint infusion averaged 13.2 (FT 28 °C) and 17.3 (FT 34 °C), which gave a result similar to the corresponding control sample. The use of the additive to sea buckthorn juice resulted in the significant deterioration of the organoleptic properties, including taste, compared to the control sample and other kvasses with additives. The highest antioxidant activity (*p* < 0.05) was demonstrated by kvass with the addition of 10% black chokeberry juice (0.734 µmol of Trolox g^−1^ (ABTS), and 4.90 µmol of Trolox g^−1^ (DPPH)) and peppermint leaf infusion (0.773 µmol of Trolox g^−1^ (ABTS) and 4.71 µmol of Trolox g^−1^ (DPPH)). The chromatographic analysis confirmed the content of 13 phenolic compounds in kvass with the addition of black chokeberry juice, which was 1.68–1.73 mg/100 mL of the finished product with a 10% share of the additive. The content of 11 phenolic compounds in kvass with the addition of peppermint infusion was confirmed and amounted to 7.65–6.86 mg/100 mL of the finished product at 10% of the additive content (*p* < 0.05). Kvasses enriched with chokeberry fruit and peppermint leaf additives may be a promising new category of functional beverages. Bioactive substances with antioxidant effects contained in these additives could contribute to improving the health-promoting properties of the tested kvasses. In the future, it is planned to conduct further research on the influence of other plant-based additives (superfruits, by-products of fruit and vegetable processing, microalgae) on the quality, nutrition, and health-promoting properties of kvass.

## Figures and Tables

**Table 1:** Criteria of organoleptic evaluation of kvass.

Points	Clarity	Smell	Color	Taste
5	very clear and uniform, slightly cloudy	very pleasant, sour, slightly yeasty	very uniform and homogeneous, very characteristic of the additives used, highly specific, intense	very pleasant, sour, yeasty, very characteristic of the flavor of the additives used
4	clear and uniform, slightly cloudy	pleasant, sour, yeasty	uniform, homogeneous, characteristic of the additives used, specific, intense	pleasant, sour, characteristic of the flavor of the additives used
3	clear and quite uniform, cloudy	quite pleasant, slightly sour	slightly less uniform and homogeneous, slightly less characteristic of additives	less pleasant, very sour, characteristic of the flavor of the additives used
2	small-colored, non-uniform	not very pleasant	slightly less uniform and homogeneous, not very characteristic of the additives used	unharmonized, too sour or too sweet, less characteristic of the additives used
1	heterogeneous, non-clarity	unpleasant, weakly perceptible	heterogeneous, uncharacteristic of the additives used	very weak or no taste at all, foreign taste, clearly altered

Own study based on Gambuś et al. [9].

**Table 2:** Results of organoleptic evaluation of kvasses with additives and control samples.

Kvass	Clarity	Smell	Color	Taste	Total Points
Fermentation Temperature 28 °C					
control kvass 28	3.8 ± 0.1 b	4.1 ± 0.2 b	3.8 ± 0.7 bc	3.4 ± 0.8 bc	15.1 ± 1.2 bc
BC (J) 3%	4.1 ± 0.3 b	4.8 ± 0.8 cd	4.9 ± 0.4 c	4.8 ± 0.4 c	18.6 ± 0.8 de
BC (J) 5%	4.3 ± 0.3 b	4.8 ± 0.9 cd	5.0 ± 0.1 c	4.8 ± 0.4 c	18.9 ± 0.6 e
BC (J) 10%	4.3 ± 0.2 b	5.0 ± 0.2 d	4.9 ± 0.2 c	4.9 ± 0.3 c	19.1 ± 0.5 e
BC (I) 3%	3.1 ± 0.8 ab	4.2 ± 0.3 b	2.1 ± 0.2 a	4.2 ± 0.3 bc	13.6 ± 0.5 b
BC (I) 5%	2.7 ± 0.7 ab	4.3 ± 0.3 b	2.2 ± 0.4 a	3.8 ± 0.7 bc	13.0 ± 0.7 b
BC (I) 10%	2.8 ± 0.7 ab	3.9 ± 0.5 b	2.2 ± 0.3 a	3.8 ± 0.8 bc	12.7 ± 0.4 b
SB(J) 3%	2.0 ± 0.2 a	2.8 ± 0.3 b	3.4 ± 0.4 b	2.1 ± 0.2 a	10.3 ± 0.3 a
SB (J) 5%	2.2 ± 0.2 a	2.7 ± 0.2 b	3.2 ± 0.2 b	1.8 ± 0.4 a	9.9 ± 0.4 a
SB (J) 10%	2.1 ± 0.3 a	3.1 ± 0.1 b	3.2 ± 0.2 b	1.8 ± 0.4 a	10.2 ± 0.6 a
SB (I) 3%	4.1 ± 0.3 b	3.0 ± 0.0 b	1.8 ± 0.3 a	3.2 ± 0.1 b	12.1 ± 0.5 a
SB (I) 5%	3.7 ± 0.5 b	3.2 ± 0.2 b	2.0 ± 0.2 a	2.1 ± 0.3 a	11.0 ± 0.5 a
SB (I) 10%	3.9 ± 0.4 b	3.2 ± 0.4 b	2.2 ± 0.4 a	2.2 ± 0.2 a	11.5 ± 0.4 a
P (I) 3%	2.8 ± 0.9 ab	4.5 ± 0.7 cd	3.3 ± 0.1 b	5.0 ± 0.4 c	15.6 ± 0.6 c
P (I) 5%	2.3 ± 0.4 a	4.7 ± 0.8 cd	3.2 ± 0.3 b	4.8 ± 0.2 c	15.0 ± 0.9 bc
P (I) 10%	2.2 ± 0.2 a	4.8 ± 0.8 cd	3.2 ± 0.3 b	4.8 ± 0.3 c	15.0 ± 0.8 bc
Fermentation Temperature 34 °C					
control kvass 34	4.7 ± 0.7 bc	4.4 ± 0.7 cd	4.0 ± 0.8 bc	4.3 ± 0.8 bc	17.4 ± 1.2 cd
BC (J) 3%	4.8 ± 0.2 c	4.9 ± 0.3 d	4.2 ± 0.9 bc	5.0 ± 0.1 c	18.9 ± 1.4 de
BC (J) 5%	5.0 ± 0.3 c	5.0 ± 0.1 d	4.8 ± 0.2 c	5.0 ± 0.1 c	19.8 ± 0.6 e
BC (J)10%	4.9 ± 0.1 c	5.0 ± 0.1 d	5.0 ± 0.4 c	5.0 ± 0.1 c	19.9 ± 0.4 e
BC (I) 3%	3.7 ± 0.4 b	4.2 ± 0.2 c	3.2 ± 0.2 b	4.1 ± 0.7 bc	15.2 ± 1.1 bc
BC (I) 5%	3.9 ± 0.2 b	4.0 ± 0.1 c	2.1 ± 0.1 a	3.7 ± 0.8 bc	13.7 ± 0.4 b
BC (I) 10%	3.3 ± 0.8 ab	4.1 ± 0.4 c	2.2 ± 0.3 a	3.9 ± 0.7 bc	13.5 ± 0.3 b
SB (J) 3%	2.1 ± 0.2 a	2.8 ± 0.4 b	3.1 ± 0.4 b	3.1 ± 0.4 b	11.1 ± 0.4 a
SB (J) 5%	2.0 ± 0.0 a	3.0 ± 0.1 b	3.3 ± 0.4 b	2.2 ± 0.2 a	10.5 ± 0.4 a
SB (J) 10%	1.8 ± 0.3 a	2.2 ± 0.4 a	3.3 ± 0.2 b	1.8 ± 0.1 a	9.1 ± 0.5 a
SB (I) 3%	4.2 ± 0.2 b	3.1 ± 0.2 b	3.2 ± 0.1 b	2.9 ± 0.2 b	13.4 ± 0.6 b
SB (I) 5%	4.0 ± 0.4 b	2.9 ± 0.3 b	3.0 ± 0.1 b	3.0 ± 0.2 b	12.9 ± 0.4 b
SB (I) 10%	3.9 ± 0.5 b	3.2 ± 0.2 ab	3.0 ± 0.2 b	3.1 ± 0.4 b	13.2 ± 0.5 b
P (I) 3%	4.2 ± 0.2 b	4.8 ± 0.4 cd	3.8 ± 0.6 bc	4.8 ± 0.3 c	17.6 ± 0.4 d
P (I) 5%	3.2 ± 0.8 ab	5.0 ± 0.1 d	3.9 ± 0.8 bc	4.9 ± 0.2 c	17.0 ± 0.8 cd
P (I) 10%	3.4 ± 0.7 ab	4.9 ± 0.4 d	4.0 ± 0.7 bc	5.0 ± 0.1 c	17.3 ± 0.9 cd

The data presented are the mean ± standard deviation (SD). Different letters in a column represent significant differences according to Tukey’s test *p* < 0.05, BC—black chokeberry; SB—sea buckthorn; P—peppermint; J—juice, I—infusion.

**Table 3:** Results of antioxidant capacity in tested kvass samples.

Factor		Total Antioxidant Activity	
Kvass (K)	Fermentation Temperature (IT)	ABTS (µmol Trolox g^−1^)	DPPH (µmol Trolox g^−1^)
Kvass (K)			
control kvass		0.264 ± 0.053 e	1.83 ± 0.57 f
BC (J) 3%		0.287 ± 0.026 f	1.75 ± 0.32 e
BC (J) 5%		0.394 ± 0.044 i	2.77 ± 0.12 j
BC (J) 10%		0.734 ± 0.046 j	3.90 ± 0.53 k
BC (I) 3%		0.160 ± 0.006 a	1.15 ± 0.13 a
BC (I) 5%		0.178 ± 0.012 b	1.40 ± 0.17 c
BC (I) 10%		0.220 ± 0.006 d	1.62 ± 0.10 d
SB (J) 3%		0.211 ± 0.015 c	1.39 ± 0.06 c
SB (J) 5%		0.262 ± 0.015 e	1.71 ± 0.07 e
SB (J) 10%		0.367 ± 0.018 h	2.21 ± 0.35 h
SB (I) 3%		0.154 ± 0.004 a	1.27 ± 0.28 b
SB (I) 5%		0.173 ± 0.005 b	1.45 ± 0.28 c
SB (I) 10%		0.226 ± 0.020 d	1.84 ± 0.54 f
P (I) 3%		0.310 ± 0.025 g	1.92 ± 0.25 g
P (I) 5%		0.399 ± 0.023 i	2.61 ± 0.05 i
P (I) 10%		0.773 ± 0.016 k	4.71 ± 0.11 l
Fermentation temperature (IT)			
28 °C		0.308 ± 0.180 a	1.99 ± 0.98 a
34 °C		0.345 ± 0.193 b	2.32 ± 1.02 b
Mean		0.321 ± 0.185	2.10 ± 1.00
Significance (F/*p* value)			
K		F= 9982.4 *p* < 0.0001	F= 5222.6 *p* < 0.0001
IT		F = 1695.5 *p* < 0.0001	F = 2594.1 *p* < 0.0001
K × IT		F = 58.3 *p* < 0.0001	F = 295.7 *p* < 0.0001

The data presented are the mean ± standard deviation (SD). Different letters in a column represent significant differences according to Tukey’s test *p* < 0.05, BC—black chokeberry; SB—sea buckthorn; P—peppermint; J—juice, I—infusion.

**Table 4:** Results of selected physicochemical indicators determining the selected tested kvass samples.

Kvass	pH Value	Alcohol Content (% vol.)	L*	a*	b*	ΔE
Fermentation Temperature 28 °C						
control kvass 28	4.10 ± 0.06 a	0.49 ± 0.04 d	40.31 ± 0.93 e	−0.30 ± 0.02 a	1.82 ± 0.04 d	na
BC (J) 5%	4.15 ± 0.01 ab	0.41 ± 0.02 d	28.55 ± 1.27 b	3.09 ± 0.23 f	−0.06 ± 0.01 a	12.4
BC (J) 10%	4.20 ± 0.01 b	0.43 ± 0.01 b	23.49 ± 1.12 a	3.36 ± 0.20 fg	−0.45 ± 0.01 b	17.5
P (I) 5%	4.39 ± 0.02 c	0.42 ± 0.01 a	36.50 ± 0.77 d	−0.81 ± 0.01 c	2.46 ± 0.03 f	3.9
P (I) 10%	4.48 ± 0.02 d	0.42 ± 0.01 a	33.31 ± 1.04 c	−1.73 ± 0.06 e	6.57 ± 0.44 i	8.7
Fermentation Temperature 34 °C						
control kvass 34	4.19 ± 0.03 ab	0.45 ± 0.02 c	39.97 ± 1.01 e	−0.24 ± 0.03 a	1.79 ± 0.02 d	na
BC (J) 5%	4.19 ± 0.02 ab	0.45 ± 0.01 c	29.04 ± 1.58 b	3.96 ± 0.08 h	1.95 ± 0.04 e	11.7
BC (J) 10%	4.25 ± 0.04 b	0.42 ± 0.02 ab	23.75 ± 1.49 a	4.01 ± 0.10 h	−1.54 ± 0.02 c	16.8
P (I) 5%	4.47 ± 0.03 d	0.40 ± 0.01 a	35.12 ± 0.89 d	−0.58 ± 0.03 b	2.97 ± 0.12 g	5.1
P (I) 10%	4.60 ± 0.04 e	0.40 ± 0.01 a	33.04 ± 0.34 c	−1.42 ± 0.03 d	5.61 ± 0.23 h	8.1

The data presented are the mean ± standard deviation (SD). Different letters in a column represent significant differences according to Tukey’s test *p* < 0.05, BC—black chokeberry; P—peppermint; J—juice, I—infusion, na—not applicable.

**Table 5:** Results of the analysis of the phenolic compounds of kvass with the addition of black chokeberry using the UPLC-PDA-MS/MS method.

Compound		Rt	λmax	[M − H] *m*/*z*			Concentration µg/100 mL		
		min	nm	MS	MS/MS	28 °C		34 °C	
						P (I) 5%	P (I) 10%	P (I) 5%	P (I) 10%
1.	Cyanidin 3-O-glucoside	2.52	279, 515	449^+^	287	59.3 b	97.7 c	48.1 a	100.8 c
2.	Cyanidin 3-O-galactoside	2.76	279, 514	449^+^	287	35.0 a	50.2 b	34.6 a	57.4 bc
3.	Neochlorogenic acid	2.26	288 sh, 324	353^−^	191	199.2 a	384.1 b	205.1 a	446.6 c
4.	Chlorogenic acid	2.86	288 sh, 324	353^−^	191	316.0 a	618.1 b	323.4 a	645.6 b
5.	Cryptochlorogenic acid	2,99	288 sh, 324	353^−^	191	52.4 b	86.4 c	46.5 ab	80.4 b
6.	Coumaroylquinic acid	3.51	309	337^−^	163	12.4 a	19.2 b	10.5 a	17.2 b
7.	Quercetin 3-O-sophoroside	3.74	255, 354	625^−^	301	21.2 a	37.2 b	21.9 a	37.5 b
8.	Kaempferol 3-O-rhamnoside-pentoside	3.81	264, 355	563^−^	285	9.5 a	18.5 b	9.7 a	18.6 b
9.	Quercetin 3-O-glucoside-pentoside	4.13	255, 354	595^−^	301	32.6 a	62.4 b	34.0 a	58.3 b
10.	Quercetin 3-O-rutinoside	4.36	255, 355	609^−^	301	31.8 a	61.4 c	34.1 a	55.9 bc
11.	Quercetin 3-O-galactoside-rhamnoside	4.44	255, 355	609^−^	301	37.0 a	67.9 b	37.9 a	62.5 b
12.	Quercetin 3-O-glucoside	4.57	255, 355	463^−^	301	46.6 a	95.4 c	47.4 a	76.8 b
13.	Quercetin 3-O-galactoside	4.71	255, 355	463^−^	301	40.7 a	81.7 c	43.2 a	69.1 b
	TOTAL					893.6 a	1680.2 b	896.3 a	1726.8 b

Different letters within a row presented significant differences according to Tukey’s test at *p* < 0.05, BC—black chokeberry; J—juice.

**Table 6:** Results of the analysis of the phenolic compounds of kvass with the addition of peppermint using the UPLC-PDA-MS/MS method.

Compound		Rt	λmax	[M − H] *m*/*z*			Concentration µg/100 mL		
		min	nm	MS	MS/MS	28 °C		34 °C	
						P (I) 5%	P (I) 10%	P (I) 5%	P (I) 10%
1.	Luteolin di-O-glucuronide	3.75	253, 347	637^−^	285	156.3 b	341.9 d	141.1 a	304.9 c
2.	Salvionolic acid H	4.31	341	537^−^	285	91.3 a	220.7 c	88.8 a	190.2 b
3.	Galloyl-gallocatechin dimer	4.57	285	761^−^	591, 305	803.4 b	1162.9 c	735.9 a	1099.0 c
4.	Quercetin 3-O-glucoside-pentoside	4.62	255, 344	595^−^	301	779.6 b	1596.7 c	682.2 a	1429.9 c
5.	Luteolin 3-O-glucuronide	4.74	253, 347	461^−^	285	331.0 a	807.9 c	296.7 a	715.6 b
6.	Salvianolic acid isomer I	5.03	285, 343	717^−^	519, 339	89.7 a	252.0 c	90.3 b	226.8 c
7.	Isorhamnetin 3-O-rutinoside	5.21	267, 336	623^−^	315	114.1 ab	230.4 d	99.5 a	201.9 c
8.	Salvianolic acid isomer II	5.32	284, 344	717^−^	519, 339	80.2 a	211.9 c	78.4 a	188.4 b
9.	Lipedoside A	5.55	251, 345	607^−^	461, 179	94.3 b	167.8 d	81.8 a	148.7 c
10.	Rosmarinic acid	5.62	329	359^−^	179	1039.4 b	2037.6 d	955.7 a	1805.6 c
11.	Sagerinic acid	5.67	283	719^−^	359, 179	322.9 b	617.2 d	286.8 a	548.9 c
	TOTAL					3902.2 b	7647.1 d	3537.3 a	6859.9 c

Different letters within a row present significant differences according to Tukey’s test *p* < 0.05, P—peppermint; I—infusion.

## Data Availability

Data is contained within the article or supplementary material.

## Supplementary Materials

The following supporting information can be downloaded at: https://www.mdpi.com/article/10.3390/foods13030357/s1, Table S1: Total antioxidant capacity in kvass samples (Kvass × Incubation Temperature).
