# Supplementary material for: Comparison of the Antioxidant and Sensorial Properties of Kvass Produced from Mountain Rye Bread with the Addition of Selected Plant Raw Materials
Source: Foods. 2024 Jan 23;13(3):357. doi: 10.3390/foods13030357 (PMC10855404; doi:10.3390/foods13030357)
Supplement: Supplementary file 1 [file foods-13-00357-s001.zip › foods-2796103-supplementary.pdf]

Table S1. Total antioxidant capacity in kvass samples (Kvass x Incubation Temperature).

Factor		Total Antioxidant Activity	
Kvass (K) x Fermentation Temperature (IT)		ABTS (µmol Trolox g^{-1})	DPPH (µmol Trolox g^{-1})
Control kvass	28°C	0.216±0.004 fgh	1.31±0.04 b
BC (J) 3%		0.271±0.006 j	1.54±0.04 ef
BC (J) 5%		0.365±0.004 n	2.70±0.06 m
BC (J) 10%		0.703±0.005 r	3.55±0.04 o
BC (I) 3%		0.157±0.004 a	1.06±0.03 a
BC (I) 5%		0.171±0.007 bc	1.29±0.04 b
BC (I) 10%		0.218±0.005 gh	1.55±0.03 ef
SB (J) 3%		0.201±0.004 ef	1.42±0.04 cd
SB (J) 5%		0.252±0.002 i	1.75±0.04 h
SB (J) 10%		0.355±0.007 mn	2.44±0.04 k
SB (I) 3%		0.152±0.004 a	1.08±0.03 a
SB (I) 5%		0.170±0.002 bc	1.26±0.02 b
SB (I) 10%		0.213±0.005 fg	1.47±0.02 de
P (I) 3%		0.294±0.009 k	1.76±0.03 h
P (I) 5%		0.384±0.008 o	2.63±0.05 lm
P (I) 10%		0.763±0.010 s	4.64±0.04 p
Control kvass	34°C	0.313±0.003 l	2.35±0.01 jk
BC (J) 3%		0.321±0.003 l	2.17±0.02 i
BC (J) 5%		0.452±0.002 q	2.91±0.08 n
BC (J) 10%		0.795±0.005 t	4.60±0.02 p
BC (I) 3%		0.166±0.004 abc	1.32±0.02 bc
BC (I) 5%		0.191±0.004 de	1.62±0.02 f
BC (I) 10%		0.225±0.005 gh	1.75±0.03 gh
SB (J) 3%		0.230±0.002 h	1.32±0.02 bc
SB (J) 5%		0.281±0.003 jk	1.62±0.02 f
SB (J) 10%		0.390±0.006 o	1.75±0.03 gh
SB (I) 3%		0.157±0.004 ab	1.64±0.04 fg
SB (I) 5%		0.179±0.003 cd	1.82±0.01 h
SB (I) 10%		0.253±0.005 i	2.56±0.05 l
P (I) 3%		0.342±0.003 m	2.25±0.03 ij
P (I) 5%		0.428±0.002 p	2.58±0.04 l
P (I) 10%		0.792±0.003 t	4.84±0.06 q
**Mean**		0.321±0.185	2.10±1.00
Significance (F / *p* value)			
K		F= 9982.4 p<0.0001	F= 5222.6 p<0.0001
IT		F= 1695.5 p<0.0001	F= 2594.1 p<0.0001
K x IT		F= 58.3 p<0.0001	F= 295.7 p<0.0001

The data shown are mean ± standard deviation (SD). Different letters within a column indicate significant The different letters in column indicate significant differences between the means at a significance level of p = 0.05, differences according to Tukey's test at p<0.05), BC - black chokeberry; SB - sea buckthorn; P – peppermint; J – juice, I – infusion.
